# The Swedish duty hour enigma

**DOI:** 10.1186/1472-6920-14-S1-S6

**Published:** 2014-12-11

**Authors:** Kristina Sundberg, Hanna Frydén, Lars Kihlström, Jonas Nordquist

**Affiliations:** 1 Department of Research and Education, Karolinska University Hospital, Stockholm, Sweden; 2 Department of Medicine, Huddinge, Karolinska Institutet, Stockholm, Sweden; 3 Department of Neurosurgery, Karolinska University Hospital, Stockholm, Sweden

## Abstract

**Background:**

The Swedish resident duty hour limit is regulated by Swedish and European legal frameworks. With a maximum average of 40 working hours per week, the Swedish duty hour regulation is one of the most restrictive in the world. At the same time, the effects of resident duty hour limits have been neither debated nor researched in the Swedish context. As a result, little is known about the Swedish conceptual framework for resident duty hours, their restriction, or their outcomes: we call this “the Swedish duty hour enigma.” This situation poses a further question: How do Swedish residents themselves construct a conceptual framework for duty hour restrictions?

**Methods:**

A case study was conducted at Karolinska University Hospital, Stockholm – an urban, research-intensive hospital setting. Semi-structured interviews were carried out with 34 residents currently in training in 6 specialties. The empirical data analysis relied on theoretical propositions and was conducted thematically using a pattern-matching technique. The interview guide was based on four main topics: the perceived effect of duty hour restrictions on (1) patient care, (2) resident education, (3) resident well-being, and (4) research.

**Results:**

The residents did not perceive the volume of duty hours to be the main determinant of success or failure in the four contextual domains of patient care, resident education, resident well-being, and research. Instead, they emphasized resident well-being and a desire for flexibility.

**Conclusions:**

According to Swedish residents’ conceptual framework on duty hours, the amount of time spent on duty is not a proxy for the quality of resident training. Instead, flexibility, organization, and scheduling of duty hours are considered to be the factors that have the greatest influence on resident well-being, quality of learning, and opportunities to attain the competence needed for independent practice.

## Background

Residents are an important part of the Swedish health care system, constituting 14% of the Swedish physician workforce [1]. Swedish residents spend a minimum of 5 years in training; all programs are outcomes-based and are derived from a common national competency framework. Residents are expected to both learn and work within the Swedish health care system [2]. The final objective is the same as elsewhere around the world: to produce physicians who are capable of competent, independent practice [3]. Table 1 provides a snapshot of the Swedish health care system.

**Table 1:** The Swedish health care system: basic facts

Total population of Sweden	9.5 million*
Total population of the capital area of Stockholm	2 050 000*

Average number of practising physicians per 1000 population	3.6†

Sources of health care financing	Taxation provides basic primary health care coverage for the entire population; 2.3% of the population have supplementary health insurance†

Primary care	Provided through public health centres: 37% of centres are run by private care companies‡

Specialist services	Provided predominantly through public hospitals†

“The Patient Care Guarantee” (*Vårdgaratin*)	A national policy aimed at providing patients with primary care within 7 days of expressed need and specialist care within 90 days of referral. In 2009-2010 the policy was successful in 87%-90% of cases§

*Statistics Sweden [4]

†Organisation for Economic Co-Operation and Development [5]

‡Swedish Competition Authority [6]

§Swedish National Board of Health and Welfare [7]

Swedish residents’ duty hours, as well as physicians’ working hours, are regulated by three legal frameworks: the European Union Working Time Directive [8], the Swedish Working Hours Act [9], and a collective agreement between the physicians’ union (the Swedish Medical Association) and the Swedish Association of Local Authorities and Regions [10]. These frameworks are summarized in Table 2.

**Table 2:** Legal frameworks for the regulation of resident duty hours in Sweden

	**European Union Working Time Directive**[8]	**Swedish Working Hours Act**[9]	**Collective Agreement (Swedish Medical Association and Swedish Association of Local Authorities and Regions)**[10]
**Weekly working hours**	Working hours must not exceed an average of 48 per week, including overtime.	Working hours must not exceed an average of 40 per week, although if overtime is necessary a maximum total of 48 working hours is permitted.	

**Rest**	A minimum daily rest period of 11 consecutive hours in every 24 hours.	A minimum daily rest period of 11 consecutive hours in every 24 hours.	

**Day/night working hours**	Extra protection in the case of night work: average working hours must not exceed 8 hours per 24-hour period.	Extra protection in the case of night work: average working hours must not exceed 8 hours per 24-hour period.	Normal working hours are between 7 a.m. and 9 p.m. Working activities outside that time frame are considered to be overtime or on-call. Alternative and flexible agreements are possible.

The introduction of the Working Time Directive by the European Union (EU) in 2003 did not have a profound impact on Sweden as an EU member state, since existing Swedish regulations were already in line with the directive. The topic of resident duty hours, their restriction, and their outcomes is seldom raised at an official level in the Swedish context, and duty hours have not been on the last two agendas of Framtidens Specialistläkare (Medical Specialists of the Future) the biennial national conference on Swedish residency training. Our review of the literature confirms there is virtually no published research originating from Sweden or elsewhere on conditions with respect to Swedish resident duty hours and their regulation [11]. There is also a lack of research on the amounts of on-call work performed by Swedish physicians and its impact on patient safety and physician well-being. The lack of research in this area is aligned with a lack of research and debate concerning working hours and workplace health and safety across all occupations in Swedish society during the last 10 years [12-15].

Restrictions for Swedish resident duty hours may appear quite restrictive from outside Swedish borders, but restrictions and the impetus behind them have different origins in different parts of the world [3]. For example, in the case of the duty-hour restrictions imposed by the Accreditation Council for Graduate Medical Education (ACGME) in the United States [16], research evidence was one of the cornerstones of the regulating body’s decision-making process [17-21]. Moreover, this process involved not only regulators but also other interest groups [3,18,22]. In parallel, research and varying perspectives on duty hour restrictions and their outcomes have been presented by other stakeholders in the United States [23-32].

However, resident duty hour restrictions do not always originate from research results and evidence. A lack of evidence and the influence of underlying assumptions [33] that are built into health care and medical education systems have been identified as drivers of the duty-hour restriction process around the world [3]. One way of describing this phenomenon is to say that different stakeholders have different conceptual frameworks [20,34] with respect to duty hours and duty hour restrictions. Conceptual frameworks in this area can be described as simplified representations of the complex relationship between duty hours and their outcomes. Such frameworks have also been described as typically implicit, such that they serve as a backdrop to research and arguments put forward about the strengths and limitations of resident duty hour restrictions [20,33].

The absence of research and debate on resident duty hours in Sweden makes it difficult to expose the underlying assumptions of the Swedish model. Our research attempted to answer the following question: How is the conceptual framework for duty hours constructed among Swedish residents?

## Method

We conducted an embedded, single case study using methods drawn from the qualitative research tradition. We use the term “case study” to refer to a study that “investigates a contemporary phenomenon in depth and within a real-life context” [35]. The “case” consisted of a group of 34 residents currently in training in 6 of the 8 main groups of specialties at the Karolinska University Hospital, a prestigious, urban, research-dominated training environment. An invitation to be a part of the case study was sent by email to all residents-in-training at this institution (*n* = 533); the first 40 who replied were included in the original intended sample. Of these, 34 (22 women and 12 men) were interviewed. Six people in the original intended sample (n = 40) turned out to have already graduated in their specialty, or did not respond when they were contacted or had the opportunity to be interviewed. Nineteen of the 34 residents were in their last year of training. Semi-structured telephone interviews were conducted with the residents during November 2011, and notes were taken in parallel by the interviewer. All interviews were conducted by a single interviewer (HF).

The interview guide was centred on four main topics, namely, the perceived effect of duty hour restrictions on (1) patient care, (2) resident education, (3) resident well-being and (4) research. The first three of these topics reflect the overarching themes have emerged from the international resident duty hour debate [19]. Our fourth theme was added because it has been observed in the Swedish context that it is difficult for residents to make time for research and doctoral education in competition with their clinical duties [36]. The topic “reported duty hours” was also included in the interview guide.

The analysis of empirical data was made with a case study approach [35]. Thus, our theoretical proposition about the notion of contextual frameworks provided a frame for our thematic analysis, which used a pattern-matching technique to compare the data from the interviews with the four predetermined thematic areas. Our aim was not to achieve traditional generalizability, by which our sample could be generalized to a wider population [37], but rather to achieve an analytical generalization, whereby our results could be generalized to a set of theories [34].

## Results

### Reported duty hours

The residents reported a wide spectrum of average weekly duty hours, with full-time work ranging from 40 to 60 hours per week. There was some variation of opinion on the part of respondents as to whether time spent on reading, administration, research, overtime, and being on-call should be counted as duty hours.

The residents reported that they found it stressful to work a higher number of duty hours, since this implied having less time for family and social commitments. They also expressed a desire for more flexibility when it came to making decisions about the amount and scheduling of duty hours. The majority of the residents could not say whether they were working too much overtime, since they had insufficient knowledge about the official regulations. Uncertainty was also expressed about what kinds of activities counted as overtime, and about whether overtime had to be requested by a supervisor, rather than performed at the resident’s discretion, in order to count as overtime.

### Patient care

The prevailing opinion among the residents was that working during the night and early morning presented a bigger threat to the quality of patient care than working too many hours. They reported that when they were tired from working nights they were less attentive and experienced a drop in their energy level, empathy with patients, and social skills. The quality of the social encounter with the patient was considered to be very important. Working in a disorganized manner was also considered a bigger potential threat to patient care than a high level of duty hours. Respondents also reported that they perceived a high level of stress in their work situation as being a more important risk factor than a high level of duty hours.

Opinions about the relationship between the volume of hours worked and the quality of patient care were ambivalent. Some respondents noted that having fewer duty hours can actually pose a threat to the quality of care by reducing opportunities to make important decisions and to gain medical and clinical knowledge. They also noted that working more hours can contribute to “flow” in the clinical situation: residents who worked longer shifts were able to contribute more information about their patients to the team as well as offering the patient a continued flow of information. Being able to influence the volume and timing of duty hours was perceived to contribute to a better working environment, a higher level of well-being for the residents, and, ultimately, a higher quality of care.

### Resident learning

A recurring theme in the resident interviews was a perception that opportunities for learning were influenced less by the *quantity* of duty hours than by the *content and organization* of duty hours. Again, working nights was not considered optimal for learning, and the residents described a fine balance between, on the one hand, not learning enough as a result of not getting enough clinical exposure, and, on the other hand, working too much and thus being tired and stressed to learn and having no time for reflection. Having sufficient, high-quality time for supervision was also considered important for learning. Some residents perceived themselves to be working mainly in “the production line,” taking care of routine cases.

The interviews suggested that a limit of 40 duty hours per week was often considered beneficial for learning and was perceived to work well with respect to achieving desired learning outcomes and competencies. However, the opportunity to be able to set aside more time for studying and reading was also sought. Feeling relaxed and content at work was also considered one of the most important factors for high-quality learning. For many respondents, being able to take care of one’s family in parallel with being a resident was a high priority. Some residents indicated that they would prefer to work more duty hours than is currently allowed, and expressed frustration about the fact that it was not possible for them to decide for themselves how much they would like to work in order to create an optimal learning environment.

### Resident well-being

The interviewees did not always perceive working a larger number of duty hours to be stressful in and of itself. Some residents perceived longer work hours as contributing to their well-being, since they found the work enjoyable, stimulating, and instructive. Instead, factors that were perceived by the residents to have a negative impact on their well-being were a fast working pace, being unable to see their families as much as they wanted, and the curtailment of their social life. Being able to exercise flexibility and make their own decisions about how many hours to work, and when, was also considered to contribute to their well-being. Still, some residents perceived a strong correlation between working too many hours, being stressed, and not feeling well. Those with families indicated that it was important to them to keep in line with the regulated duty hour limit in order to achieve a good balance between family life and work.

### Research

A common opinion among the residents was that clinical work often got in the way of research. They indicated that the clinical department demands a certain level of clinical work, and sometimes prohibits residents from setting aside time for research, even though they felt pressured to pursue research activities. The residents would have preferred to have longer periods of continuous research time. Some residents, however, were reluctant to take time off for research, fearing that doing so would impede the development of their clinical competence. The residents reported that research activities were possible only if they worked at night, during weekends, while on parental leave, and so on, and that pursuing research also prolonged the time needed to complete residency training.

## Discussion

**Figure 1:**
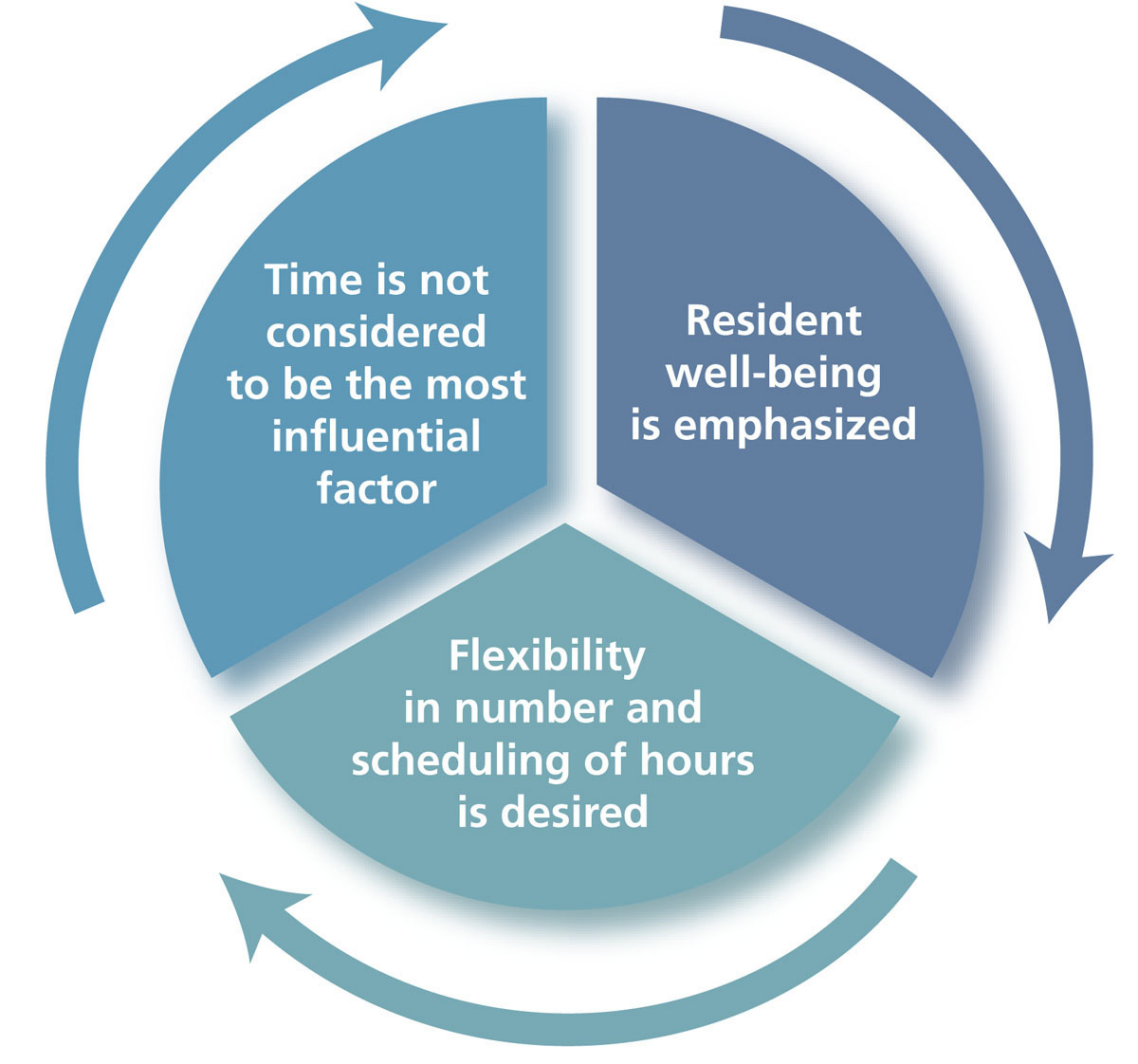
Resident duty hours: the Swedish conceptual framework

The residents’ conceptual framework for duty hours was characterized by the following themes (Fig. 1):

• a rejection of the assumption that the volume of duty hours was always the most important determinant of success or failure in the four conceptual framework domains

• an emphasis on resident well-being

• a desire for flexibility (i.e., the ability to determine the number and timing of one’s own duty hours)

The structure of the residents’ contextual framework regarding duty hours can also be seen as a reflection of the social contract between Swedish physicians and the public. This social contract is dependent on the balance between society’s expectations of the profession, and the professions’ own view of its mission [38]. Hence, the contextual framework expresses features of the Swedish residents’ view of their professional role, which must in turn be accepted by the Swedish public to be maintained.

Resident well-being was the predominant theme in the residents’ conceptual framework for duty hours, and also had a bearing on the themes of patient care and resident learning. Again, well-being was portrayed as closely connected to one’s family situation; indeed, it becomes rather evident that the typical Swedish resident is at an age where he or she may have a young family. In Sweden, both parents work full-time to a large extent: 93% of fathers and 66% of mothers with children at home work full time [39]. The notion of well-being is, again, also closely connected to flexibility: to have the freedom to choose when to work and how work is organized was considered more important than how many hours they had to work. This is confirmed by the theories of Karasek and Theorell, which describe how a greater degree of active control and decision-making capacity in the workplace can attenuate the stress created by workplace demands [40]. In this model, residency training would be described as presenting high psychological demands. A perceived low level of control over the flexibility of duty hours can potentially contribute to psychological strain and physical illness. On the other hand, having greater flexibility with respect to duty hours could potentially increase residents’ motivation for learning [40]. Finnish research on municipal employees has shown that flexible working hours lead to lower levels of sick leave [41]. Again, the emphasis on well-being and on family and social life as being central to the residents’ values indicates a harmony in the Swedish social contract. The residents probably emphasized the same things that the general public would emphasize as being important in their daily lives. The Swedish public expects residents to work the same number of hours as themselves in a common context, which is embodied in the Swedish Working Hours Act [10].

With regard to patient care, the quality of the social encounter with the patient seemed to be more in the consciousness of the residents than the issue of patient safety and its relationship to the volume of duty hours. This, also, appears to be in alignment with the social contract between the public and the medical profession in Sweden, where proportionately few cases of physician malpractice are reported [15]. Also, no data are collected in the Swedish health care system on patient satisfaction and experiences. Sweden has no formal definition of patients’ rights at a governmental level, and hospitals are not required to have a means of registering patients’ complaints. Although it is possible, in the event of a medical error, for a patient to take the case to court [5], this is not standard procedure. One could also highlight the fact that research has shown a linkage between high-quality patient communication and the absence of reported malpractice claims [42,43]. The Swedish Patient Safety Law was not established until 2010 [44].

Resident learning was also not perceived by the residents as being exclusively influenced by the number of duty hours worked. The residents’ well-being, social factors such as a balanced family life, and flexibility in the work situation were all factors brought forward as important for optimal learning. The fact that Swedish residents do perceive it to be necessary or even desirable to work too long at night to achieve optimal learning also indicates something about the social contract with the public. Even though independent practice is, of course, the optimal outcome for the Swedish public as well as for the profession, the social contract does not seem to include an expectation that residency is a professional rite of passage during which the residents’ stamina will be tested [17]. A high level of performed duty hours, especially at night, did not seem to be perceived as a necessary indicator of a “strong” resident.

### Limitations of the study

Sweden’s restrictions on resident duty hours are among the strictest in the world, and expressions such as “high” and “low” in the residents’ description of their level of duty hours should be interpreted in the context of that standard. Still, the residents in our study reported that they occasionally worked more than the allowable number of hours. However, our study does not reveal the exact number of duty hours that Swedish residents perform on average; answering this question was not the main aim of our research, nor was it aligned with our study design. To obtain data on the exact number of performed duty hours, a quantitative methodology would be preferable. It would also take a larger, national sample to conduct such mapping with any validity. Our study, therefore, does not support conclusions about the value of the current Swedish regulation of duty hours.

Further, this case study does not reveal whether activities that are believed to exceed the regulated duty hour limit can indeed be counted as such. For future research, it would be important to define the exact criteria for activities that are officially accepted on a Swedish national level as constituting “duty hours,” since this did not seem to be very clear among our interviewees. However, Swedish residency training programs are not accredited at a national level [45]; in the absence of a regulating body that monitors the levels of duty hours in Sweden, it would be difficult to expose any trend among residents of exceeding the regulated maximum. Also, it would not be in the interest of employers to expose such a situation, since they are dependent on the work capacity of the residents. Nor would it be in the interest of residents who make additional money from working overtime to disclose that they are working beyond the regulated hours [14].

Other limitations of our study should be pointed out. For example, although our findings may provide some insight into the current situation, they cannot necessarily be generalized to Sweden as a whole. It is also important to bear in mind that the selection of interviewees depended on the residents’ active consent to be interviewed. Further, our study focuses solely on the conceptual framework of residents; to obtain a richer picture, future research should also examine the perceptions of other stakeholders. Other limitations of the study include the fact that our analyses rely heavily on empirical data from residents’ own perceptions and self-reporting and do not include direct observations of the residents’ working conditions.

## Conclusions

Approaches to duty hour regulation should always be discussed with a view to cultural and social contexts. The Swedish model, viewed internationally as a strict standard for the restriction of resident duty hours, is the result of a traditionally strong national union movement and a culturally specific social contract between the Swedish public and the medical profession. The restrictive nature of duty hour regulation in Sweden is not threatening to the social contract between medical professionals and the Swedish public; instead, it is aligned with the Swedish public’s expectations of residents and physicians. Moreover, the residents in our case study did not appear to perceive the 40-hour limit as a threat to their objective to become independent practitioners after the completion of training. Having said this, it is evident that some residents did feel that not being able to exceed the regulated duty hour limit inhibited their learning opportunities. Future research on this perception, as well as objective data on the average number of duty hours actually worked by Swedish residents, is needed.

Judging from the results of this case study, in the Swedish system the number of duty hours performed is not considered a proxy for the quality of residency training and its various components. Instead, the factors perceived to have the greatest influence on residents’ well-being and, ultimately, the quality of learning and attainment of competence for independent practice are flexibility, organization, and degree of influence on the scheduling of duty hours.

Residency training always occurs in a specific cultural context. By highlighting the Swedish residents’ conceptual framework on duty hours, this study can nuance the international debate on duty hours and their regulation. Sharing other examples and thus challenging the conceptual frameworks developed and nurtured in specific cultures and contexts enables a richer picture to appear.

## Authors’ contributions

KS designed and coordinated the study, participated in the analysis and interpretation of data and drafted the manuscript. HF collected the data, participated in the interpretation of data and helped to draft the manuscript. LK participated in the design of the study and helped to draft the manuscript. JN participated in the design of the study and the interpretation of data, and helped to draft the manuscript. All authors read and approved the final manuscript.

## Competing interests

The authors have no competing interests to declare.

## Declarations

Resources and secretariat support for this project was provided by the Royal College.

This article has been published as part of *BMC Medical Education* Volume 14 Supplement 1, 2014: Resident duty hours across borders: an international perspective. The full contents of the supplement are available online at http://www.biomedcentral.com/bmcmededuc/supplements/14/S1. This article was submitted in 2011 and peer reviewed in 2012. Final acceptance for publication as part of this supplement was in 2014.

Publication of this supplement was supported and funded by the Royal College of Physicians and Surgeons of Canada. The funding agency played no role in the design, in the collection, analysis and interpretation of data; in the writing of the manuscripts; and in the decision to submit the manuscript for publication. The articles have been through the journal's standard peer review process for supplements. The Supplement Editors declare that they have no competing interests.
